# Impact of hypoxia on the hippocampus: A review

**DOI:** 10.1097/MD.0000000000041479

**Published:** 2025-03-21

**Authors:** Guan Lu, Ge Rili, Ma Shuang

**Affiliations:** a Research Center for High Altitude Medicine, School of Medicine, Qinghai University, Xining, China; b Key Laboratory for Application for High Altitude Medicine, Qinghai University, Xining, China.

**Keywords:** BDNF, hippocampus, hypoxia, learning and memory, neuron

## Abstract

Oxygen is the most abundant chemical substance and is a basic material for human activities. A decline in oxygen concentration affects many physiological processes in the body, leading to pathological changes and even the occurrence of diseases. Therefore, an increasing number of studies have focused on the pathological state of hypoxia. The hippocampus is the most sensitive tissue to oxygen in the brain. The reduction in oxygen concentration affects the morphology and functioning of the hippocampus, including a decline in learning and memory, immunity, and energy metabolism, causing great problems to people’s physical and mental health. To keep people healthy in hypoxic environments, adapt to hypoxic environments, and avoid diseases, it is necessary to review the morphology and function of the hippocampus, as well as the effect of oxygen on the hippocampus.

## 1. Introduction

Hypoxia can be divided into acute, chronic, persistent, intermittent, chemical, and postpartum hypoxia based on the cause of the disease. Hypoxic preconditioning and treatment can also be used to treat diseases. Hypoxia is a major pathological factor in brain injury.^[1]^ The hippocampus is the most sensitive tissue in the brain to hypoxia, and hypoxia affects attention, executive function, learning and memory, speed of memory processing, declarative memory, and other functions of the hippocampal tissue.^[2,3]^ Different degrees and types of hypoxia have different effects on the hippocampus. Some studies have proposed that transient hypoxia can cause structural changes and dysfunction of neurons, including the shortening and thinning of dendrites and weakening of excitatory synaptic transmission strength, which affect brain development.^[4]^ This article reviews the effects of hypoxia on hippocampal learning and memory, neuronal development and angiogenesis, synaptic plasticity, inflammation and immunity, oxidative stress, autophagy, apoptosis, and energy metabolism.

## 2. Morphology and function of hippocampus

**Figure 1.**
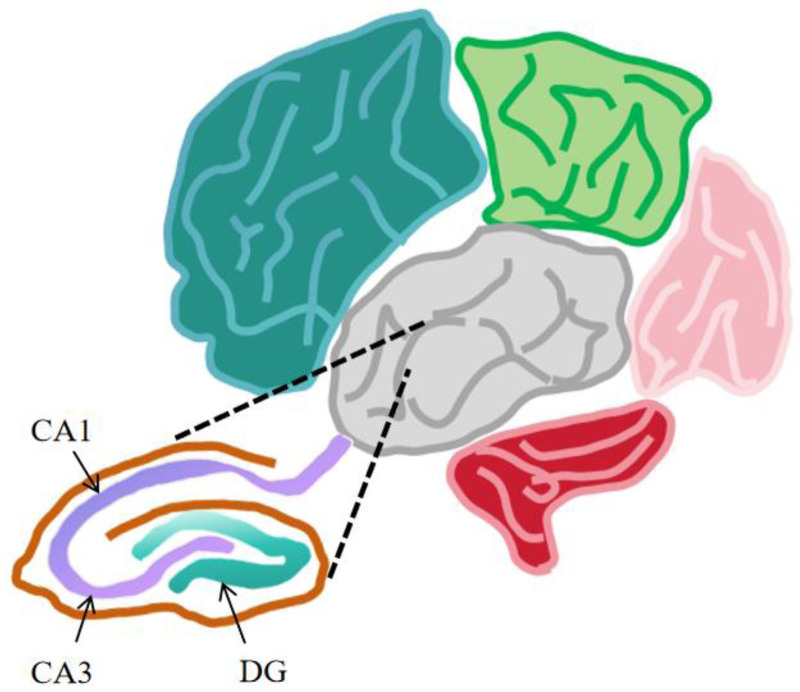
The structure of the hippocampus in the brain

The hippocampus, also known as the hippocampal gyrus, hippocampal area, or brain hippocampus, is named for its structural shape, which is similar to that of the hippocampus. Located between the cerebral colliculus and medial temporal lobe, the hippocampal formation is composed of the dentate gyrus and inferior torus of the hippocampus and its adjacent temporal lobe, which can be divided into CA1, CA2, CA3, and DG areas, which belong to a part of the limbic system (Fig. 1).

Currently, the role of the hippocampus remains controversial, but most scientists believe that the hippocampus has episodic or autobiographical memories. The hippocampus is not only involved in the formation of new memories but also in learning and emotion. There are also some fixed-point cells in the hippocampus, which are involved in the storage and processing of spatial information. When information enters the hippocampus, it first passes through the CA3 area, then the CA1 area, and then reaches the subthalamus.

Damage to the hippocampus can affect memories and the ability to form new memories. Spatial memory is the ability to remember direction, position, and orientation. Damage to the left hippocampus affects the memory of language information, whereas damage to the right hippocampus affects the memory of visual information.

## 3. Effects of hypoxia

### 3.1. Learning and memory and cognitive function

Hypoxia impairs the cognitive and motor abilities of mice by affecting hippocampal mitochondrial oxidative phosphorylation but has no significant effect on the morphology and structure of the brain in mice.^[5]^ The learning and memory abilities of rats were also affected by hypoxia, which showed that the escape latency of rats was prolonged, damage to hippocampal neurons was reduced, and learning and memory abilities were restored to some extent. In addition, hypoxia-induced hippocampal atrophy, memory impairment, and decreased motor responses in rats.^[6]^ Hypoxia promotes tau phosphorylation through the extracellular regulated protein kinases (ERK) signaling pathway, which affects learning and memory.^[7]^ Hypoxia promotes the expression of matrix metalloprotein 9 in the rat hippocampus and affects passive avoidance learning abilities in rats.^[8]^ Hypoxia affects horizontal movement and carding activity in rats. Rats show differences in different areas of hypoxia, and the results also differ according to sex.^[1]^ In a hypoxic environment of 4010 m in the hippocampus, the latency and number of crossings of the salidroside platform in mice were reduced, the damage to hippocampal neurons was alleviated, the expression of apoptotic proteins was reduced, and the learning and memory ability was improved.^[9]^

Acute high-altitude hypoxic exposure can change the morphology of vertebral neurons in the hippocampal CA1 area, affect the expression of proteins in the hippocampus, and lead to cognitive dysfunction.^[10]^ Under continuous hypoxia, the dendritic spines in the hippocampal CA1 region of mice decreased, synaptic plasticity decreased, escape latency was prolonged, and spatial learning and memory abilities decreased.^[11]^ After acute high-altitude exposure for 7 days, mice showed obvious memory impairment, hippocampal neuron pyknosis, and increased expression of apoptotic proteins.^[12]^ Exposure to acute hypobaric hypoxia exposure can impair cognitive function in rats. Ginkgolide B intervention may regulate hippocampal neuronal calcium homeostasis by regulating the hippocampal Calcium/calmodulin-dependent protein kinase type Ⅱ signaling pathway, alleviating hippocampal neuronal injury, improving learning and spatial memory in rats, and alleviating cognitive dysfunction.^[13]^

The effects of hypobaric hypoxia on the nervous system have become increasingly serious over the years. When exposed to hypoxia for 2 days, the dendritic length of pyramidal cells decreased and was the most serious on the 7th day. One of the mechanisms underlying the hippocampal-dependent spatial learning decline is dendritic cell atrophy.^[14]^ Single-simulated hypobaric hypoxia is beneficial to the behavior of mice by activating mild stress in the mitochondria.^[15]^ Hypobaric hypoxia impairs the cognitive, motor coordination, and spatial memory ability of rats by inhibiting the synthesis of catecholamine,^[16]^ also available via peroxisome proliferator-activated receptor-gamma coactivator (PGC)-1alpha/fibronectin type III domain-containing protein 5/brain-derived neurotrophic factor (BDNF) signaling pathway causes damage to hippocampal mitochondria and synapses, leading to memory impairment.^[17]^ Hypobaric hypoxia can improve oxidative stress and synaptic plasticity by activating the Notch pathway, which can cause learning and memory impairment and reduce the cognitive index of new objects in mice.^[18]^

Chronic exposure to hypoxia at high altitudes results in a reduction in hippocampal gray matter volume, impaired learning and memory ability, and anxiety-like behavior in rats.^[19]^ Chronic intermittent hypoxia leads to anxiety-like behavior in mice, which is related to the compensatory increase in NMDA receptor 2B, ERK, and the synaptic plasticity of neurons.^[20]^ Chronic intermittent hypoxia can reduce synaptic plasticity in hippocampal neurons by activating adenosine A2AR, resulting in spatial memory impairment in mice. Knocking out or inhibiting adenosine A2AR improves synaptic plasticity in hippocampal neurons and reduces spatial memory impairment in mice.^[21]^ Chronic intermittent hypoxia can promote the expression of ERK1/2 in the hippocampus, reduce the volume of hippocampal neurons, pyknosis of nucleus, cause cognitive dysfunction, increase the escape latency, and reduce the number of crossing the platform. Chronic intermittent hypoxia promotes the expression of inflammatory factors in the hippocampus, leading to microglia damage and cognitive decline.^[22]^ Intermittent hypoxia caused the loss of NMDA receptor-dependent long-term enhancement and the increase of reactive oxygen species (ROS) in the hippocampal CA1 region, resulting in the decrease of hippocampal synaptic plasticity and the impairment of spatial memory ability, which was related to hypoxia-inducible factor (HIF-1) alpha (HIF-1α) dependent redox state changes.^[23]^ Intermittent hypoxia through the nuclear factor kappa-B (NF-κB) signaling pathway causes neuronal apoptosis, affects cognitive function of mice, and prolongs the latency of target quadrant in mice.^[24]^ Intermittent hypoxia therapy promotes tau phosphorylation through the mammalian target of rapamycin (mTOR) signaling pathway, reduces the expression of glutamate-relevant proteins in the hippocampus, and improves cognitive function in mice.^[25]^ Intermittent hypoxia promotes oxidative stress in rat hippocampal neurons and affects learning and memory. This situation can be improved using the total glycosides of Cistanche deserticola.^[26]^

Prenatal hypoxia interferes with hippocampal CA3-CA1 synaptic transmission and reduces long-term potentiation, which may be caused by the inhibition of GluN2B protein expression, ultimately leading to serious damage to the learning and memory ability of rats.^[27]^ It can also reduce the number of hippocampal neurons and damage long-term memory.^[28]^ Ischemia and hypoxia can inhibit the expression of BDNF and glialcellline-derivedneurotrophic factor, leading to hippocampal atrophy,^[29]^ neurological dysfunction, and learning and memory impairment in rats.^[30]^ Hypercapnia combined with hypoxemia can improve the nuclear translocation efficiency of HIF-1α in hippocampal neurons, promote the expression of matrix metalloproteinases, damage the blood-brain barrier in the hippocampus, and lead to cognitive dysfunction.^[31]^ Hypoxic preconditioning downregulated the expression of miR-103, reduced the incubation period, increased the number of crossings, and improved hypoxic tolerance and cognitive function in mice.^[32]^

### 3.2. Neurons and blood vessels

Hypoxia at high altitudes led to the widening of the gap around nerve cells, swelling of cells, atrophy and deformation of neurons, disordered arrangement, and loss of neurons in the hippocampal CA1 region of rats.^[33]^ Hypoxia reduces the proliferation and differentiation of hippocampal dentate gyrus neurons in mice, which may be related to the ERK signaling pathway.^[34]^ Repeated hypoxic exposure reduced the metabolic rate and promoted the expression of immediate early gene products in hippocampal neurons.^[35]^ Hypoxia increases the expression of neurodegenerative genes in hippocampal neurons.^[36]^ Hypoxia increases the number of proximal dendrites of hippocampal CA1 pyramidal neurons and prolongs the branches of apical dendrites, affecting certain functions of hippocampal neuronal circuits.^[37]^ Hypoxic exposure promotes the growth of hippocampal neuronal axons and enhances the reverse transport function of mitochondrial axons, which may be achieved by promoting the expression of the mitochondrial transport regulatory protein HUMMR.^[38]^ The decrease in calcium accumulation in hippocampal CA1 neurons during hypoxia may be related to the enhanced hypoxia tolerance in hippocampal neurons caused by long-term hypoxia.^[39]^ Hypoxia improves the permeability of the blood-brain barrier through the ERK signaling pathway.^[40]^ After the administration of kaempferol, the learning and spatial memory abilities of rats were restored, and neuronal degeneration was reduced.^[41]^ Egg-laying defective nine 1 regulates the hypoxic response of hippocampal neurons by regulating the transcriptional activity of HIF-1α. Therefore, inhibiting expression of Egg-laying defective nine 1 has a protective effect on hippocampal neurons.^[42]^ Hypoxia combined with propofol can induce iron death, mitochondrial swelling, cristae dissolution, and disappearance in hippocampal neurons by promoting the expression of the transferrin receptor and ferrous ions, further aggravating the damage of immature hippocampal neurons.^[43]^ Astragalus membranaceus protected hippocampal neurons from intermittent hypoxia-induced injury in rats.^[44]^ The expression of synaptophysin in the hippocampus decreased, and the expression of vascular endothelial growth factor increased in the hypoxia group.^[45]^ Hypoxia disrupts the formation of hippocampal capillaries. The older the age, the lower the plasticity of hippocampal capillaries.^[46]^

Acute high-altitude hypoxia can promote the necrosis of pyramidal neurons in the hippocampal CA1 area, mitochondrial swelling, and the appearance of a large number of mitochondrial autophagosomes, leading to the apoptosis of hippocampal neurons. This mechanism may be regulated by the phosphatidylinositol-3-kinase (PI3K)/protein kinase B/mTOR signaling pathway.^[47]^ After the AAV-syn-BDNF-Enhanced green fluorescent protein virus was transferred into hippocampal neurons and treated with acute hypobaric hypoxia, the spontaneous bioelectrical activity of the neurons was restored, the anti-hypoxia ability was improved, and neuropathy was reduced.^[48]^ Acute high-altitude hypoxia reduces cerebral blood flow and damages the central nervous system of mice.^[49]^

Chronic hypobaric hypoxia exposure leads to increased neuronal degeneration in the hippocampal CA3 region, which is related to the regulation of tropomyosin receptor kinase B. Hypoxia-induced memory impairment leads to decreased spatial memory in adult rats by reducing the immunoreactivity of the postsynaptic density protein kalirin-7.^[50]^ Intermittent hypoxia can promote hippocampal functions, including cell proliferation, migration of newborn neurons, and increased spinous processes, which may be related to notch1-mediated neurogenesis.^[51]^ Intermittent hypoxia inhibits the development of adult hippocampal neurons, impairs the spatial memory ability of animals, and increases the generation of adult neurons after the termination of hypoxia. HIF-1α in intermittent hypoxia-dependent hippocampal neurons is activated in early neurons and promotes the formation of adult neurons after removing hypoxia conditions.^[52]^ Intermittent hypoxia leads to decreased growth of growth hormone and vascular endothelial growth factor (VEGF) in the hippocampus, resulting in neuronal loss and cognitive impairment.^[53]^ Chronic intermittent hypoxia reduces synaptic plasticity by inhibiting the protein kinase A signaling pathway, leading to neuronal damage.^[54]^ Intermittent hypoxia promotes neuronal deformation, nuclear membrane blurring, and mitochondrial vacuolation in the rat hippocampal CA1 region. Edaravone increases and reduces the expression of apoptotic proteins and autophagy in hippocampal neurons and plays a neuroprotective role.^[55]^

Hypoxic ischemia promotes the expression of hypoxia-inducible factor-1, HIF-1α, which leads to decreased systolic and diastolic blood pressure and mean arterial pressure in rats. After treatment with salidroside, hemodynamic indices improved. This result is consistent with the regulation of HIF-1α protein.^[56]^ Ischemic hypoxia can affect the survival rate of hippocampal neurons and promote neuronal degeneration by promoting the expression of HIF-1α.^[57]^ The expression of HIF-1α positive cells increased in the hippocampus of rats in the hypoxia group, and the density of VEGF-positive cells was higher than that of the control group.^[58]^ Studies have found that early inhibition of the Toll-like receptor 4 (TLR4) signaling pathway can increase the density of the spinal cord in the hippocampus, improve the loss of neurons, and decrease synaptic plasticity caused by hypoxic-ischemic injury.^[59]^ Ischemia and hypoxia reduce cerebral perfusion, affect brain metabolism, and damage hippocampal neurons in oligodendrocyte nuclei.^[60]^ Prenatal hypoxia reduced reelin expression in the rat hippocampus but did not affect VEGF expression.^[61]^ Hypoxia induced by hypercapnia reduces the expression of proteins involved in neuronal stratification and migration in the hippocampal CA1 region, particularly in the hippocampal pyramidal cell layer.^[62]^ Hypoxia and hypoglycemia can reduce the synaptic plasticity of hippocampal neurons, resulting in reduced cAMP-response element binding protein (CREB) and BDNF expression. After using jitianjiannao, the CREB/BDNF pathway is activated, which enhances the expression of synaptic plasticity proteins, promotes synaptic remodeling, and has a protective effect on hippocampal neurons in rats.^[63]^ The administration of sodium pyruvate under hypoxic conditions can activate autophagy signaling and reduce apoptosis in hippocampal neurons, thus playing a neuroprotective role and reducing hypoxic injury.^[64]^ Hypoxic preconditioning can improve cell viability and protect hippocampal neurons from hypoxia-induced injury.^[65]^ Intermittent preconditioning for 6 h per day can effectively improve the number of remaining hippocampal neurons and tissue ultrastructure, which is related to the regulation of signaling pathways, including the endocytosis signaling pathway, longevity regulation pathway, and RNA transport.^[66]^

### 3.3. Synaptic morphology, function, and plasticity

Hypoxia exposure inhibits the expression of developmental proteins in hippocampal neurons, including the hypoxia-related molecules fibronectin 1 and filament protein C, ultimately leading to neuronal damage.^[67]^ The expression of synaptic plasticity proteins in hippocampal CA1 neurons was significantly decreased, the number of synapses in the neurons was reduced, and the synaptic gap was unclear. With the extension of intermittent hypoxia time, synaptic damage becomes more serious.^[68]^ Hypoxia leads to an abnormal increase in glutamate levels, shortening of neurites, imbalance in internal homeostasis, and memory impairment.^[69]^ The level of extracellular γ-aminobutyric acid (GABA) in hippocampal synaptosomes of rats exposed to hypoxia increased.^[70]^

High-altitude hypoxia exposure can reduce the myelin sheath content and decrease the thickness of the myelin sheath in the corpus callosum and DG area of the hippocampus in adult mice. Reoxygenation can return to normal within 2 months, and hypoxic reoxygenation does not affect axon content.^[71]^ High-altitude hypoxia induces a decrease in the density of dendritic spines in hippocampal CA1 neurons in mice. After using inhibitors, the morphological changes in dendritic spines in hippocampal CA1 neurons were protected, and damaged learning and memory abilities were restored.^[72]^ Butylphthalide pretreatment can increase acetylcholine and acetylcholine activity and reduce the damage caused by acute hypobaric hypoxia on memory function in mice.^[73]^ Inhibition of adenosine by hypoxia can lead to neuronal damage, behavioral changes in the hippocampal CA1 area, and impaired memory.^[74]^

Chronic hypoxia affects myelination and motor coordination in adult mice.^[75]^ Hypoxia for 30 min reduced excitatory postsynaptic potential and synaptic transmission of evoked fields in the hippocampal CA1 region.^[76]^ Reversible synaptic inhibition caused by hypoxia for 40 minutes was transformed into the irreversible disappearance of the synaptic potential through an NMDAR-dependent mechanism. This phenomenon occurs in the presence of plasma 1, and the presence of a transport inhibitor cannot block the effect mediated by plasma 1, leading to the accumulation of unknown transporters, cell swelling, and further damage to hippocampal neurons.^[77]^ Short-term hypoxia can damage GABAergic neurons and reduce their inhibitory effects, which may be achieved through the PI3K signaling pathway.^[78]^ Continuous hypoxia affects synaptic dendritic spine density in the hippocampal CA1 region of senescence accelerated mouse male mice and reduces synaptic plasticity. After treatment with dihydrotestosterone, the expression of the synaptic plasticity proteins in the hippocampus of mice increased.^[79]^ Studies have found that ligustrazine hydrochloride has a protective effect on the learning and memory abilities of the brain under hypobaric hypoxia, and its mechanism may be related to an increase in the expression of forkhead box p2 in the hippocampus of rats.^[80]^

Chronic hypobaric hypoxia exposure inhibited the expression level of fillin in the hippocampal CA1 region of mice. It resulted in morphological changes in dendritic spines in the hippocampal CA1 region, including an increase in the length of dendritic spines and apical dendritic spines.^[81]^ Chronic high-altitude hypoxia can damage the hippocampal neurons, promote apoptosis, affect oxidative stress, and free radical levels, and lead to cognitive dysfunction. One of these mechanisms involves increased glutamate and receptor-mediated excitotoxicity.^[82]^

Chronic intermittent hypoxia can damage cholinergic neurons in the basal forebrain through endoplasmic reticulum stress, oxidative stress, and inflammatory reactions and cause cognitive dysfunction in mice. The basal forebrain cholinergic system can play a role in endoplasmic reticulum stress, oxidative stress, and inflammatory reactions reduce damage to cholinergic neurons, and thus restore cognitive function in mice.^[83]^ Severe chronic intermittent hypoxia results in an increase in the number of GABA neurons in the hippocampus of mice, and the number of GABA neurons in males was higher than that in females.^[84]^ Chronic intermittent hypoxia inhibits the mTOR/NF-κB signaling pathway, leading to decreased BDNF-mediated synaptic plasticity and cognitive impairment.^[85]^ After intermittent hypoxia and continuous hypoxia, the activity of carbachol in the DG region of the rat hippocampus decreases, which may be due to hypoxia inhibiting the activity of the G protein in the DG region and affecting the function of the hippocampus.^[86]^

Ischemic hypoxia leads to neurodegeneration by damaging the CA1 synaptic transmission and cell integrity.^[87]^ Ischemic hypoxia inhibits the expression of glutamate and aspartic acid in the CA1 region of the hippocampus, leading to an increase in the number of damaged neurons.^[88]^ Hypoxic ischemia inhibits the synaptic plasticity of hippocampal neurons, inhibits Pro BDNF to BDNF transformation, and leads to brain injury by downregulating the BDNF/TrkB pathway.^[89]^ Convulsions induced by ischemia and hypoxia change the purinergic and neuroinflammatory components in an age-dependent manner in the developing mouse hippocampus.^[90]^ Perinatal hypoxia affects synaptic plasticity and cognitive function and leads to long-term biological dysplasia.^[91]^ Perinatal ischemia and hypoxia can reduce the expression of hippocampal interneurons, such as somatostatin and neuropeptide Y, and affect brain development^[92]^ and the release of GABA in the nerve endings of the hippocampal tissue and damage to the hippocampus.^[28]^ Repeated hypercapnia and hypoxia increase the expression of the acetylcholine receptor subunit in the hippocampus, but the increase in the CA1 area was not obvious.^[93]^

### 3.4. Immunity and inflammation

High altitude and low-pressure hypoxia can increase the expression of adenosine A2A receptor and tumor necrosis factor-α (TNF-α) in rat hippocampal brain, leading to the accumulation of microglia, mediating the occurrence of neuroinflammation, and leading to acute spatial memory impairment in mice.^[94]^ Hypobaric hypoxia causes significant damage to the hippocampal CA1 region. Hypoxia activates astrocytes and microglia, increases pro-inflammatory factor levels, and induces neurodegeneration.^[95]^ With the increase of hypoxia altitude and time, the PI3K/Akt/mTOR-HIF-1α signal pathway was gradually inhibited. Verbascoside can alleviate damage to hippocampal tissue and oxidative stress in vivo and has a certain protective effect on the cognitive function damage caused by hypoxia at high altitudes.^[96]^ Hypoxic exposure can promote the expression of inflammatory factor TNFα in the hippocampus of mice and inhibit the expression of neuroprotective genes such as BDNF and CREB in the hippocampus; however, Genistein can reverse the situation.^[97]^ Hypoxia induces an inflammatory reaction in HT22 cells. After treatment with dexmedetomidine, the level of MMP relatively increased. TNF-α expression level was relatively low, which inhibits the occurrence of inflammatory reaction.^[98]^ After moderate hypoxia, the number of immunopositive neurons in the dorsal hippocampus and abdomen increased, which may have been caused by the activation of intermediate neurons containing neuropeptide Y.^[99]^

Acute hypoxia can promote the activation of microglial type M1 and reduce the activation of microglial type M2, accompanied by an increase in related pro-inflammatory cytokines and chemokines and a decrease in anti-inflammatory cytokines.^[100]^ Acute hypobaric hypoxia exposure promotes hippocampal TLR-4, and Tongxinluo intervention can inhibit TLR4/Myeloid differentiation primary response protein 88/NF-κB. Activating the B signaling pathway can reduce the expression of the above inflammatory factors, reduce the inflammatory reaction in the hippocampus, and improve brain edema.^[101]^

Chronic intermittent hypoxia induces inflammation both in vivo and in vitro in mice, leading to inflammatory responses and neuronal apoptosis in the hippocampus. The SUMO-specific protease reverse inflammatory responses.^[102]^ Chronic intermittent hypoxia promotes the activation of microglia and expression of pro-inflammatory factors in the rat hippocampus, impairs the learning and memory abilities of rats, and affects cognitive function.^[103]^ Chronic intermittent hypoxia via NF-κB-mediated c-Jun N-terminal kinase signaling pathway promotes the expression of inflammatory factors in the hippocampus, which leads to severe oxidative stress in the hippocampus and impairs cognitive function.^[104]^ Chronic intermittent hypoxia promotes the production of hippocampal TNFα and interleukin-1 β. Propofol significantly improved cognitive function in mice, possibly related to its inhibition of hippocampal inflammatory response.^[105]^ Chronic intermittent hypoxia promotes the expression of inflammatory factors in the hippocampus, induces an oxidative stress response, and leads to depression-like behavior in rats.^[106]^ Intermittent hypoxia promoted malat1 and NOD-, LRR- and pyrin domain-containing protein 3 (NLRP3) expression in hippocampal neurons, which was negatively correlated with the expression of mir-224-5p. MALAT1 can affect the expression of NLRP3 by regulating mir-224-5p, while up-regulating mir-224-5p can reduce the inflammatory activation of microglia and ultimately regulate NLRP3/IL-1 in the hippocampus.^[107]^ IH-induced cognitive impairment is closely related to oxidative stress injury in hippocampal neurons. Compared with the CA3 region, CA1 region is more vulnerable to oxidative stress. Edaravone can reduce hippocampal damage by clearing excess ROS to normalize the oxidative balance.^[108]^ Intermittent hypoxia induces inflammatory factors P2X7 receptor(P2X7R) and TNFα in the rat hippocampus; when the concentration of P2X7R is increased and the expression of P2X7R is inhibited, the transformation from M1 to M2 type microglia is inhibited, and the expression level of pro-inflammatory factors in the hippocampus is decreased.^[109]^ Intermittent hypoxia promotes the expression of neuroinflammatory factors and inflammatory changes in microglia of the dorsal hippocampus by affecting cognitive function in mice.^[110]^ Intermittent hypoxia promotes Peroxisome proliferator-activated receptor γ post-translational modification and can cause inflammation and neuronal apoptosis, leading to cognitive impairment.^[111]^ Intermittent hypoxia leads to oxidative stress injury and cognitive impairment in mice; Peroxisome proliferator-activated receptor γ pioglitazone, an agonist, can improve oxidative stress injury and cognitive impairment caused by intermittent hypoxia.^[112]^

Hypercapnia can promote the activation of the NLRP3 inflammasome in microglia activated by hypoxia and pro-il-1 (Cut to IL-1), which can then induce the inflammatory reaction of the central nervous system, leading to the apoptosis of hippocampal neurons and aggravating the damage to cognitive function.^[113]^ After hypoxic preconditioning, the expression of NF-kB and phosphorylated CREB increases in the dentate gyrus of rats, increasing immune reactivity and brain tolerance to hypoxia.^[114]^ After hypoxia and reoxygenation, the expression of inflammatory genes in the hippocampus increases.^[115]^

### 3.5. Oxidative stress, apoptosis, and autophagy

Hypoxia for 6h in HT22 mouse hippocampal cells promoted PGRN expression, leading to brain injury.^[116]^ Hypoxia-induced oxidative stress in the hippocampus promotes memory and cognitive impairments, leading to neurodegeneration.^[117]^ Hypoxia increases lactate dehydrogenase (LDH) and intracellular calcium overload, reduces total superoxide dismutase activity, increases malondialdehyde levels, and promotes oxidative stress and apoptosis.^[118]^ The antioxidant system in the brain was damaged, and the cells were apoptotic when rats were exposed to 7% oxygen for 6 hours. Compared to the cortex, apoptosis of hippocampal neurons is more obvious.^[119]^ Hypoxia for 18 hours promotes the expression of HIF-1α, leading to loss of mitochondrial membrane potential, nerve cell rupture, and apoptosis.^[120]^ Hypoxia inhibits BDNF expression and reduces the anti-apoptotic ability of hippocampal neurons.^[121]^ The learning and memory abilities of rats are impaired in natural high-altitude hypoxic environments. As mitochondrial damage in hippocampal neurons increases, neuronal apoptosis increases and nerve regeneration decreases.^[122]^ Hypoxia can also increase the unfolded protein response, promote apoptotic signal transduction, and increase tau phosphorylation, leading to cell dysfunction.^[123]^ After treatment with melatonin, malondialdehyde expression and apoptosis in the hippocampus decreased.^[122]^ Hypoxia promotes the combination of HIF-1α and hypoxia response elements, impairs the hypoxia tolerance of NMR hippocampal neurons, and induces apoptosis of hippocampal neurons. Studies have found that after 8 h of hypoxia, the protein in hippocampal neurons increased, which may be caused by the increased expression of HIF-1α, further reducing the apoptosis of neurons caused by hypoxia.^[123]^ Hypoxia enhances the migration of neural stem cells, causes cell death, and contributes to the survival of newborn cells in the hippocampus.^[124]^ The damage caused by hypoxia to hippocampal neurogenesis and cell death can be reversed by melatonin, which may be through inhibition of the activation of hippocampal NF-κB.^[125]^ The autophagic activity of the hippocampal neurons was relatively low. When rats are exposed to hypoxia, the protein level of autophagy markers in the hippocampus is low, which indicates the vulnerability of hippocampal CA1 neurons to some extent. In the hippocampus, the exposure to hypoxia resulted in a decreased autophagy marker, which was followed by activation of the autophagy-related gene expressions.^[126]^

Hypobaric hypoxia exposure promotes the expression of apoptotic proteins in the hippocampus, which may be related to the increased expression of c-Jun N-terminal kinase, and the depletion of keratin 18 may be a possible mechanism.^[127]^ Hypobaric hypoxia promotes the leakage of LDH and the expression of apoptotic proteins, leading to oxidative stress, excitotoxicity of glutamate, and neurodegeneration.^[128]^

Chronic intermittent hypoxia promotes the apoptosis of hippocampal neurons and affects the spatial learning and memory abilities of rats.^[129]^ Intermittent hypoxia promotes the expression of superoxide dismutase in hippocampal neurons, leads to oxidative stress and apoptosis, causes spatial reference and working memory disorders in rats, and leads to cognitive impairment.^[130]^ Intermittent hypoxia increases the expression of apoptotic proteins in the hippocampus and prolongs the escape latency in rats, suggesting that their learning and spatial memory abilities are impaired.^[131]^ Intermittent hypoxia promotes autophagy in hippocampal neurons, resulting in chromatin concentration, cell fragmentation, and a reduced quantity of hippocampal neurons.^[132]^

After 6 hours of sublethal hypoxia, the synthesis of nitric oxide synthase increased, leading to cell necrosis in the hippocampus and cortex.^[133]^ Ischemia and hypoxia lead to developmental disorders of hippocampal neurons, characterized by hippocampal atrophy, decreased neuronal count, and increased cell apoptosis. Hypoxia ischemia injury leads to increased expression of key molecules regulating apoptosis in the hippocampal CA1 region, resulting in apoptosis of hippocampal astrocytes and further leading to neuronal degeneration.^[134]^ After moderate focal ischemia and hypoxia, many hippocampal neurons were damaged and atrophied. Light microscopy shows that dead neurons are similar to apoptotic neurons.^[135]^ Hydrogen sulfide protects rat hippocampal neurons from hypoxia-reoxygenation (H/R) injury by promoting RhoA phosphorylation of ser188 and reducing the leakage of LDH and neuron-specific enolase (NSE). After hypoxic reoxygenation, cell viability is damaged, resulting in the leakage of LDH and NSE.^[136]^ Sodium cyanide induces chemical ischemia and hypoxia in hippocampal neurons by promoting the production of prostaglandin G/H synthase 2, resulting in increased hippocampal neuronal nuclear fragmentation and decreased cell viability. Hydroxygentine exerts neuroprotective effects by inhibiting the production of prostaglandin G/H synthase 2.^[137]^ Hypoxic preconditioning can improve hypoxia tolerance, maintain the stability of hippocampal formation, and protect the hippocampus from hypoxic damage by activating endogenous antioxidant defense systems.^[138]^ Hypoxic reoxygenation for 6 h induces oxidative stress in cells, leading to apoptosis, which is related to a decrease in miR-200 family expression.^[139]^ Hypoxic reoxygenation can also damage the mitochondrial membrane potential in the hippocampus, resulting in increased ROS production and decreased antioxidant capacity.^[140]^

### 3.6. Mitochondria and metabolism

Hypoxia reduces the rate of mitochondrial oxygen consumption induced by ADP in the mouse hippocampus, whereas a ketogenic diet increases the rate of oxygen consumption and the expression of mitochondrial division and fusion proteins in the mouse hippocampus.^[141]^ Under mild hypoxia, neurons may reduce apoptosis of hippocampal neurons by upregulating lactate dehydrogenase A expression.^[142]^ Hypoxia causes mitochondrial dysfunction, increased excitotoxicity, and neurodegeneration in rat hippocampal neurons, which may be related to the regulation of mitochondrial biogenesis by ERK-nuclearfactor erythroidderived 2-like 2.^[143]^ Hypoxia combined with hypercapnia promotes the expression of proteins related to the permeability regulation of blood-brain barrier in the hippocampus, leading to the destruction of blood-brain barrier in SD rats.^[144]^ Rhodiola rosea can resist hypoxic injury by inhibiting the opening of the mitochondrial membrane transport pore, preventing changes in mitochondrial membrane potential and the difference in Ca^2+^ concentration inside and outside the mitochondrial membrane, increasing the expression of Bcl-2, reducing the expression of Caspase3, and inhibiting neuronal apoptosis.^[145]^ In addition, hypoxia can lead to reduced expression of glycolytic genes, such as glycerate kinase 1, in the hippocampal tissue, and its expression decreases with age.^[2]^ Hypoxia leads to lipid peroxidation and DNA damage in the hippocampal neurons, and the molecular chaperone heat shock protein 90 is involved in this pathological process.^[146]^ Severe hypoxia can promote hippocampal DNA fragmentation and reduce thiobarbituric acid-reactive substances, whereas hypoxic postconditioning can inhibit these phenomena and protect hippocampal neurons.^[147]^ Under severe hypobaric hypoxia, the expression of glucose-6-phosphate dehydrogenase and the levels of NADPH and total glutathione in the rat hippocampus decreased. After post-treatment, the activity of glucose-6-phosphate dehydrogenase was restored, and the levels of NADPH and total glutathione increased slowly, reducing neuronal death of neurons.^[148]^ Curcumin intervention can improve the decrease in dendritic spine density, synaptic damage, and learning and memory impairment of hippocampal neurons induced by hypoxia by regulating phenylalanine metabolism, glucose metabolism, and bile acid synthesis in the hippocampus of hypoxia-exposed mice.^[149]^

Acute hypobaric hypoxia leads to an increase in lipid peroxide content and free radicals in hippocampal neurons.^[150]^ Acute high altitude hypoxia affects the level of energy metabolism in rats, which shows that the mitochondrial membrane potential of hippocampal neurons, mitochondrial respiratory chain complexes Ⅰ, Ⅲ and Ⅳ and ATPase activity are significantly decreased. After well point bloodletting, the above situation was improved, which may be related to the reduction of mitochondrial autophagy level mediated by PI3K-Akt-mTOR signaling pathway.^[151]^

Chronic hypoxia leads to the formation of immature dendritic cells by altering energy metabolism and neurotransmitter transmission in the hippocampus, which affects fear memory in the hippocampus.^[152]^ Chronic hypobaric hypoxia exposure promotes the expression of aging proteins in the hippocampus, inhibits tau expression, and leads to a decline in learning and memory abilities in rats. Changes in the protein metabolism of the hippocampal mitochondrial morphology lead to lipofuscin accumulation, which further causes the degeneration of hippocampal CA3 neurons.^[153]^

Chronic intermittent hypoxia can lead to the damage of hippocampal neurons in rats, resulting in the serum HIF-1α. NSE concentration increased; however, with the extension of hypoxia time, serum HIF-1α expression increased after the intervention of Dazhu Hongjingtian, and the damage of hippocampal neurons was improved.^[154]^ Chronic intermittent hypoxia results in a decrease in body weight and hippocampal weight in rats and metabolic changes in the hippocampal tissue, including a decrease in glutamate levels.^[155]^ Studies have found that chronic intermittent hypobaric hypoxia can alleviate the decline in mitochondrial membrane potential and outflow of the mitochondrial apoptosis promoter protein cytochrome c during cerebral ischemia.^[156]^ Huperzine A alleviates chronic intermittent hypoxia-induced hippocampal neuronal apoptosis, oxidative stress, and synaptic plasticity damage by reducing iron deposition in mouse brains.^[157]^ Intermittent hypoxia promotes the generation of oxygen free radicals, reduces the number of mitochondria, and affects the morphology and structure of the hippocampus in rats.^[158]^ Intermittent hypoxia leads to the damage of multiple cognitive domains in the hippocampus, and the level of substance metabolism in the hippocampus increases, suggesting that the damage of cognitive domains may be related to the loss of hippocampal function.^[159]^ Intermittent hypoxia affects the level of oxidative bases in hippocampal mitochondrial DNA (mtDNA) and the expression of key enzymes for mitochondrial base excision repair at the gene level and significantly reduces the contents of total mtDNA1 and mtDNA3.^[160]^

Hypoxic preconditioning changes the metabolic patterns in the serum and hippocampus of mice, and the metabolism of glutathione, alanine, aspartate, and glutamate in the hippocampus changes significantly.^[161]^ Hypoxia preconditioning enhances the expression of Thioredoxin-2 in hippocampal mitochondria, which may be a reactive regulator of hypoxia.^[162]^ Hypoxic preconditioning exerts neuroprotective effects by affecting energy metabolism in hippocampal cells through the mTOR/autophagy signaling pathway.^[163]^ The main source of NADPH in the brain is the pentose phosphate pathway, which is involved in glucose metabolism. Studies have found that Timely supplementation with NADPH after ischemia or hypoxia can improve oxidative stress, apoptosis, and neuronal loss in the hippocampus.^[164]^ Hypoxic reoxygenation promotes the expression of apoptosis-inducing factor, mitochondrial division-related proteins and Rho a protein 1 and damages hippocampal neurons.^[165]^ Hypoxia treatment can promote lipopolysaccharide to increase the level of chemokine ligand 10 in the serum and hippocampus of mice, and its mechanism is related to NF-kB signaling pathway activation.^[166]^

## 4. Conclusions

**Figure 2.**
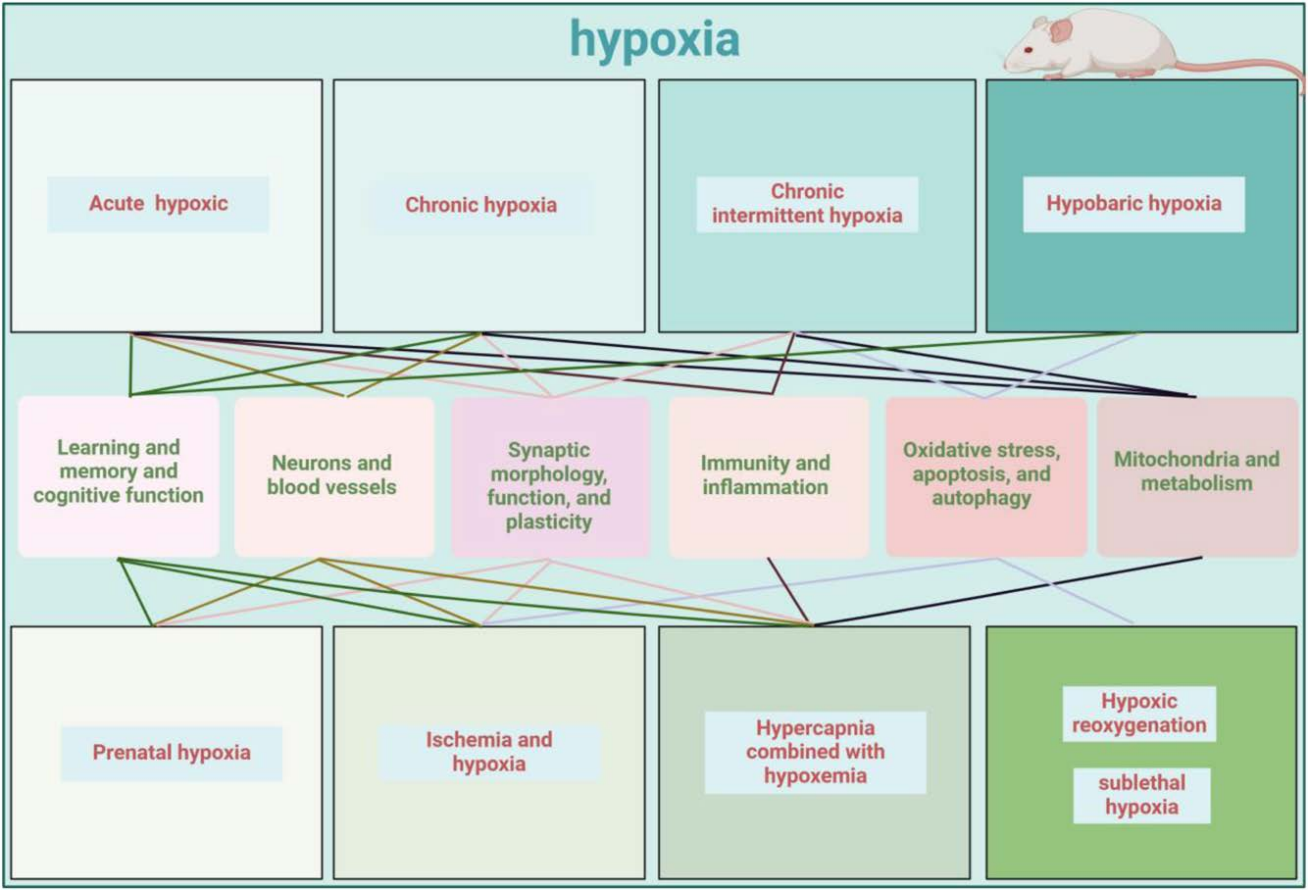
Impact of hypoxia on the hippocampus. Various types of hypoxia affect the morphology and function of the hippocampus to varying degrees, and ultimately affect the body.

Changes in oxygen concentration affect the hippocampal morphology and structure. Morphological and functional changes in the hippocampus are affected by hypoxia type, hypoxia time, and oxygen concentration. Hypoxia affects hippocampal learning and memory ability, oxidative stress level, neuron development, and metabolism (Fig. 2). Further intervention and the discovery of therapeutic targets will help stabilize hippocampal function under hypoxia. It is expected that in the future, there will be more means to treat the consequences of abnormal changes in the hippocampus under hypoxic environments and develop an effective defense.

## Acknowledgments

These figures were created with BioRender.com.

## Author contributions

**Conceptualization:** Ge Rili.

**Funding acquisition:** Ma Shuang, Ge Rili.

**Investigation:** Ma Shuang.

**Methodology:** Ma Shuang.

**Validation:** Ge Rili.

**Writing – original draft:** Guan Lu.

**Writing – review & editing:** Guan Lu.

## References

[1] Lin Y Liu X Tan D Jiang Z. Atractylon treatment prevents sleep-disordered breathing-induced cognitive dysfunction by suppression of chronic intermittent hypoxia-induced M1 microglial activation. Biosci Rep. 2020;40:BSR20192800. 32490526 10.1042/BSR20192800 PMC7295624

[2] Alam S Ray K Jain V. Reduced expression of Kalirin-7 contributes to working memory deficit during chronic hypobaric hypoxia exposure. Behav Brain Res. 2019;366:135–41. 30851319 10.1016/j.bbr.2019.03.016

[3] Alvarez-Merz I Luengo JG Munoz MD Hernandez-Guijo JM Solis JM. Hypoxia-induced depression of synaptic transmission becomes irreversible by intracellular accumulation of non-excitatory amino acids. Neuropharmacology. 2021;190:108557. 33848510 10.1016/j.neuropharm.2021.108557

[4] Andresen JH Loberg EM Wright M Goverud IL Stray-Pedersen B Saugstad OD. Nicotine affects the expression of brain-derived neurotrophic factor mRNA and protein in the hippocampus of hypoxic newborn piglets. J Perinat Med. 2009;37:553–60. 19492919 10.1515/JPM.2009.081

[5] Arias-Cavieres A Khuu MA Nwakudu CU Barnard JE Dalgin G Garcia AR. A HIF1a-dependent pro-oxidant state disrupts synaptic plasticity and impairs spatial memory in response to intermittent hypoxia. eNeuro. 2020;3:7. 10.1523/ENEURO.0024-20.2020 PMC7363479 32493757

[6] Bhattacharjee M Manoharan S Deshetty UM Perumal E. Acute hypobaric hypoxia exposure causes neurobehavioral impairments in rats: role of brain catecholamines and tetrahydrobiopterin alterations. Neurochem Res. 2023;48:471–86. 36205808 10.1007/s11064-022-03767-x

[7] Biswal S Sharma D Kumar K. Global hypoxia induced impairment in learning and spatial memory is associated with precocious hippocampal aging. Neurobiol Learn Mem. 2016;133:157–70. 27246251 10.1016/j.nlm.2016.05.011

[8] Boissard CG Lindner MD Gribkoff VK. Hypoxia produces cell death in the rat hippocampus in the presence of an A1 adenosine receptor antagonist: an anatomical and behavioral study. Neuroscience. 1992;48:807–12. 1630626 10.1016/0306-4522(92)90268-7

[9] Cai J Ruan J Shao X. Oxygen enrichment mitigates high-altitude hypoxia-induced hippocampal neurodegeneration and memory dysfunction associated with attenuated tau phosphorylation. High Alt Med Biol. 2021;3:274–84. 10.1089/ham.2020.0218 34348049

[10] Car H Michaluk P. Baclofen influences acquisition and MMP-2, MMP-9 levels in the hippocampus of rats after hypoxia. Pharmacol Rep. 2012;3:536–45. 10.1016/s1734-1140(12)70849-6 22814007

[11] Celik K Bilim P Garip G Durmaz B Yildirim SE Baka M. Acute hypoxia exposure following prenatal stress impairs hippocampus and novelty-seeking behavior in adolescent rats. Int J Dev Neurosci. 2022;1:85–95. 10.1002/jdn.10162 34850973

[12] Chatzi C Schnell E Westbrook GL. Localized hypoxia within the subgranular zone determines the early survival of newborn hippocampal granule cells. Elife. 2015;4:e8722. 10.7554/eLife.08722 PMC4714973 26476335

[13] Chen C Li B Chen H. Epigallocatechin-3-gallate ameliorated iron accumulation and apoptosis and promoted neuronal regeneration and memory/cognitive functions in the hippocampus induced by exposure to a chronic high-altitude hypoxia environment. Neurochem Res. 2022;8:2254–62. 10.1007/s11064-022-03611-2 PMC9352632 35552996

[14] Chen PZ He WJ Zhu ZR. Adenosine A(2A) receptor involves in neuroinflammation-mediated cognitive decline through activating microglia under acute hypobaric hypoxia. Behav Brain Res. 2018;347:99–107. 29501623 10.1016/j.bbr.2018.02.038

[15] Churilova AV Rybnikova EA Glushchenko TS Tiul’Kova EI Samoilov MO. Effect of mild hypobaric hypoxia in the preconditioning regime on expression of pCREB and NF-kappaB transcription factors in the rat hippocampus before and after severe hypoxia. Morfologiia. 2009;6:38–42. 20358771

[16] Churilova A Zachepilo T Baranova K Rybnikova E. Differences in the autophagy response to hypoxia in the hippocampus and neocortex of rats. Int J Mol Sci . 2022;23:8002. 35887346 10.3390/ijms23148002 PMC9320385

[17] Coimbra-Costa D Alva N Duran M Carbonell T Rama R. Oxidative stress and apoptosis after acute respiratory hypoxia and reoxygenation in rat brain. Redox Biol. 2017;12:216–25. 28259102 10.1016/j.redox.2017.02.014 PMC5334548

[18] Cui J Ma Q Zhang C. Keratin 18 depletion as a possible mechanism for the induction of apoptosis and ferroptosis in the rat hippocampus after hypobaric hypoxia. Neuroscience. 2023;513:64–75. 36395917 10.1016/j.neuroscience.2022.11.009

[19] Das D Biswal S Barhwal KK Chaurasia OP Hota SK. Kaempferol inhibits extra-synaptic NMDAR-mediated downregulation of TRkbeta in rat hippocampus during hypoxia. Neuroscience. 2018;392:77–91. 30266684 10.1016/j.neuroscience.2018.09.018

[20] Despotovski V Vivekanandarajah A Waters KA Machaalani R. Early postnatal exposure to intermittent hypercapnic hypoxia (IHH), but not nicotine, decreases Reelin in the young piglet hippocampus. Neurotox Res. 2022;6:1859–68. 10.1007/s12640-022-00598-0 PMC9797456 36322363

[21] Dheer A Jain V Kushwah N Kumar R Prasad D Singh SB. Temporal and spatial changes in glial cells during chronic hypobaric hypoxia: role in neurodegeneration. Neuroscience. 2018;383:235–46. 29751055 10.1016/j.neuroscience.2018.04.026

[22] Dhillon SK Gunn ER Pedersen MV. Alpha-adrenergic receptor activation after fetal hypoxia-ischaemia suppresses transient epileptiform activity and limits loss of oligodendrocytes and hippocampal neurons. J Cereb Blood Flow Metab. 2023;6:947–61. 10.1177/0271678X231153723 PMC10196751 36703575

[23] Dong P Zhao J Li N. Sevoflurane exaggerates cognitive decline in a rat model of chronic intermittent hypoxia by aggravating microglia-mediated neuroinflammation via downregulation of PPAR-gamma in the hippocampus. Behav Brain Res. 2018;347:325–31. 29574103 10.1016/j.bbr.2018.03.031

[24] Fan Y Chou MC Liu YC Liu CK Chen CH Chen SL. Intermittent hypoxia activates N-methyl-D-aspartate receptors to induce anxiety behaviors in a mouse model of sleep-associated apnea. Mol Neurobiol. 2021;58:3238–51. 33660202 10.1007/s12035-021-02321-0

[25] Fang H Zhang LF Meng FT Du X Zhou JN. Acute hypoxia promote the phosphorylation of tau via ERK pathway. Neurosci Lett. 2010;3:173–7. 10.1016/j.neulet.2010.03.037 20304032

[26] Gao H Han Z Huang S. Intermittent hypoxia caused cognitive dysfunction relate to miRNAs dysregulation in hippocampus. Behav Brain Res. 2017;335:80–7. 28647595 10.1016/j.bbr.2017.06.025

[27] Ghotbeddin Z Basir Z Jamshidian J Delfi F. Modulation of behavioral responses and CA1 neuronal death by nitric oxide in the neonatal rat’s hypoxia model. Brain Behav. 2020;10:e1841. 10.1002/brb3.1841 PMC7667332 32940009

[28] Gonzalez FJ Insausti SR Cebada SS. Neuropeptides in the developing human hippocampus under hypoxic-ischemic conditions. J Anat. 2021;4:856–68. 10.1111/joa.13458 PMC8450465 34028021

[29] Gorgias N Maidatsi P Tsolaki M. Hypoxic pretreatment protects against neuronal damage of the rat hippocampus induced by severe hypoxia. Brain Res. 1996;714:215–25. 8861628 10.1016/0006-8993(95)01548-5

[30] Guan R Yang C Zhang J Wang J Chen R Su P. Dehydroepiandrosterone alleviates hypoxia-induced learning and memory dysfunction by maintaining synaptic homeostasis. CNS Neurosci Ther. 2022;28:1339–50. 35703574 10.1111/cns.13869 PMC9344085

[31] Hambrecht VS Vlisides PE Row BW Gozal D Baghdoyan HA Lydic R. Hypoxia modulates cholinergic but not opioid activation of G proteins in rat hippocampus. Hippocampus. 2007;17:934–42. 17598161 10.1002/hipo.20312

[32] Hota KB Hota SK Chaurasia OP Singh SB. Acetyl-L-carnitine-mediated neuroprotection during hypoxia is attributed to ERK1/2-Nrf2-regulated mitochondrial biosynthesis. Hippocampus. 2012;22:723–36. 21542052 10.1002/hipo.20934

[33] Hota SK Hota KB Prasad D Ilavazhagan G Singh SB. Oxidative-stress-induced alterations in Sp factors mediate transcriptional regulation of the NR1 subunit in hippocampus during hypoxia. Free Radic Biol Med. 2010;49:178–91. 20381604 10.1016/j.freeradbiomed.2010.03.027

[34] Howell KR Pillai A. Long-term effects of prenatal hypoxia on schizophrenia-like phenotype in heterozygous reeler mice. Mol Neurobiol. 2016;53:3267–76. 26059812 10.1007/s12035-015-9265-4

[35] Hung MW Tipoe GL Poon AM Reiter RJ Fung ML. Protective effect of melatonin against hippocampal injury of rats with intermittent hypoxia. J Pineal Res. 2008;44:214–21. 18289174 10.1111/j.1600-079X.2007.00514.x

[36] Ingraham JP Forbes ME Riddle DR Sonntag WE. Aging reduces hypoxia-induced microvascular growth in the rodent hippocampus. J Gerontol A Biol Sci Med Sci. 2008;63:12–20. 18245756 10.1093/gerona/63.1.12

[37] Iwai T Obara K Ito C Furukawa H Oka JI. Hydroxyobtustyrene protects neuronal cells from chemical hypoxia-induced cell death. J Nat Med. 2018;72:915–21. 29987461 10.1007/s11418-018-1224-8

[38] Jain V Baitharu I Barhwal K Prasad D Singh SB Ilavazhagan G. Enriched environment prevents hypobaric hypoxia induced neurodegeneration and is independent of antioxidant signaling. Cell Mol Neurobiol. 2012;4:599–611. 10.1007/s10571-012-9807-5 PMC11498567 22331403

[39] Ji W Zhang Y Ge RL Wan Y Liu J. NMDA receptor-mediated excitotoxicity is involved in neuronal apoptosis and cognitive impairment induced by chronic hypobaric hypoxia exposure at high altitude. High Alt Med Biol. 2021;1:45–57. 10.1089/ham.2020.0127 33252277

[40] Jun YH Ju GS Chung YY. Differential expression of vascular endothelial growth factor in the cortex and hippocampus upon cerebral hypoperfusion. In Vivo. 2020;1:191–7. 10.21873/invivo.11761 PMC6984084 31882479

[41] Khuu MA Nallamothu T Castro-Rivera CI Arias-Cavieres A Szujewski CC Garcia IA. Stage-dependent effects of intermittent hypoxia influence the outcome of hippocampal adult neurogenesis. Sci Rep. 2021;1:6005. 10.1038/s41598-021-85357-5 PMC7966401 33727588

[42] Kim MJ Lim HS Yoo YB. Expression of CD95 and CD95L on astrocytes in the CA1 area of the immature rat hippocampus after hypoxia-ischemia injury. Comp Med. 2007;6:581–9. 18246871

[43] Krugers HJ Maslam S Korf J Joels M Holsboer F. The corticosterone synthesis inhibitor metyrapone prevents hypoxia/ischemia-induced loss of synaptic function in the rat hippocampus. Stroke. 2000;5:1162–72. 10.1161/01.str.31.5.1162 10797181

[44] Lanigan S Corcoran AE Wall A Mukandala G O’Connor JJ. Acute hypoxic exposure and prolyl-hydroxylase inhibition improves synaptic transmission recovery time from a subsequent hypoxic insult in rat hippocampus. Brain Res. 2018;1701:212–8. 30244114 10.1016/j.brainres.2018.09.018

[45] Li MM Wu LY Zhao T. The protective role of 5-hydroxymethyl-2-furfural (5-HMF) against acute hypobaric hypoxia. Cell Stress Chaperones. 2011;16:529–37. 21494793 10.1007/s12192-011-0264-8 PMC3156263

[46] Li RC Guo SZ Raccurt M. Exogenous growth hormone attenuates cognitive deficits induced by intermittent hypoxia in rats. Neuroscience. 2011;196:237–50. 21888951 10.1016/j.neuroscience.2011.08.029 PMC3260052

[47] Li W Yang S Yu FY. Hydrogen ameliorates chronic intermittent hypoxia-induced neurocognitive impairment via inhibiting oxidative stress. Brain Res Bull. 2018;143:225–33. 30243887 10.1016/j.brainresbull.2018.09.012

[48] Ling J Yu Q Li Y. Edaravone improves intermittent hypoxia-induced cognitive impairment and hippocampal damage in rats. Biol Pharm Bull. 2020;8:1196–201. 10.1248/bpb.b20-00085 32475934

[49] Liu D Wang Z Zhan J. Hydrogen sulfide promotes proliferation and neuronal differentiation of neural stem cells and protects hypoxia-induced decrease in hippocampal neurogenesis. Pharmacol Biochem Behav. 2014;116:55–63. 24246910 10.1016/j.pbb.2013.11.009

[50] Liu G Zhao W Zhang H Wang T Han Z Ji X. rs1769793 variant reduces EGLN1 expression in skeletal muscle and hippocampus and contributes to high aerobic capacity in hypoxia. Proc Natl Acad Sci USA. 2020;47:29283–5. 10.1073/pnas.2010073117 PMC7703601 33109725

[51] Liu P Zou D Yi L. Quercetin ameliorates hypobaric hypoxia-induced memory impairment through mitochondrial and neuron function adaptation via the PGC-1alpha pathway. Restor Neurol Neurosci. 2015;2:143–57. 10.3233/RNN-140446 25588463

[52] Liu X Ding H Li X. Hypercapnia exacerbates the blood-brain barrier disruption via promoting HIF-1a nuclear translocation in the astrocytes of the hippocampus: implication in further cognitive impairment in hypoxemic adult rats. Neurochem Res. 2020;7:1674–89. 10.1007/s11064-020-03038-7 PMC7224048 32328929

[53] Lu Y Wang J Tang F. Regulation and role of neuron-derived hemoglobin in the mouse hippocampus. Int J Mol Sci. 2022;10:5360. 10.3390/ijms23105360 PMC9140924 35628182

[54] Luo L Lu L Lu Y. Effects of hypoxia on progranulin expression in HT22 mouse hippocampal cells. Mol Med Rep. 2014;5:1675–80. 10.3892/mmr.2014.2016 24604062

[55] Mitroshina Capital IEC Mishchenko TA Usenko AV. AAV-Syn-BDNF-EGFP virus construct exerts neuroprotective action on the hippocampal neural network during hypoxia in vitro. Int J Mol Sci. 2018;8:2295. 10.3390/ijms19082295 PMC6121472 30081596

[56] Naganuma S Ota K. Continuous intensive peritoneal dialysis. Nihon Rinsho. 1991;49:538–44. 1808314

[57] Nathaniel TI Otukonyong E Abdellatif A Soyinka JO. Effect of hypoxia on metabolic rate, core body temperature, and c-fos expression in the naked mole rat. Int J Dev Neurosci. 2012;30:539–44. 22633996 10.1016/j.ijdevneu.2012.04.004

[58] Orso R Creutzberg KC Lumertz FS. Early environmental enrichment rescues memory impairments provoked by mild neonatal hypoxia-ischemia in adolescent mice. Behav Brain Res. 2021;407:113237. 33798820 10.1016/j.bbr.2021.113237

[59] Papazisis G Pourzitaki C Sardeli C Lallas A Amaniti E Kouvelas D. Deferoxamine decreases the excitatory amino acid levels and improves the histological outcome in the hippocampus of neonatal rats after hypoxia-ischemia. Pharmacol Res. 2008;57:73–8. 18243015 10.1016/j.phrs.2007.12.003

[60] Peterson BL Larson J Buffenstein R Park TJ Fall CP. Blunted neuronal calcium response to hypoxia in naked mole-rat hippocampus. PLoS One. 2012;7:e31568. 22363676 10.1371/journal.pone.0031568 PMC3283646

[61] Pokorny J Trojan S. Chronic changes in the receptive field of the pyramidal cells of the rat hippocampus after intermittent postnatal hypoxia. Physiol Bohemoslov. 1983;5:393–402. 6647587

[62] Pozdnyakova N Yatsenko L Parkhomenko N Himmelreich N. Perinatal hypoxia induces a long-lasting increase in unstimulated gaba release in rat brain cortex and hippocampus. The protective effect of pyruvate. Neurochem Int. 2011;58:14–21. 20970472 10.1016/j.neuint.2010.10.004

[63] Riljak V Lastuvka Z Myslivecek J Borbelyova V Otahal J. Early postnatal hypoxia induces behavioral deficits but not morphological damage in the hippocampus in adolescent rats. Physiol Res. 2020;1:165–79. 10.33549/physiolres.934234 PMC8565955 31852194

[64] Rodriguez-Alvarez N Jimenez-Mateos EM Engel T. Effects of P2X7 receptor antagonists on hypoxia-induced neonatal seizures in mice. Neuropharmacology. 2017;116:351–63. 28082183 10.1016/j.neuropharm.2017.01.005

[65] Rognlien A Wolle EJ Atneosen-Asegg M Suganthan R Bjoras M Saugstad OD. Neonatal Ogg1/Mutyh knockout mice have altered inflammatory gene response compared to wildtype mice in the brain and lung after hypoxia-reoxygenation. J Perinat Med. 2018;1:114–24. 10.1515/jpm-2018-0172 30020889

[66] Rubin BR Milner TA Pickel VM. Sex and age differentially affect GABAergic neurons in the mouse prefrontal cortex and hippocampus following chronic intermittent hypoxia. Exp Neurol. 2020;325:113075. 31837319 10.1016/j.expneurol.2019.113075 PMC7050962

[67] Rumman M Pandey S Singh B Gupta M Ubaid S Mahdi AA. Genistein prevents hypoxia-induced cognitive dysfunctions by ameliorating oxidative stress and inflammation in the hippocampus. Neurotox Res. 2021;39:1123–33. 33740236 10.1007/s12640-021-00353-x

[68] Rzemieniec J Litwa E Wnuk A. Neuroprotective action of raloxifene against hypoxia-induced damage in mouse hippocampal cells depends on ERalpha but not ERbeta or GPR30 signalling. J Steroid Biochem Mol Biol. 2015;146:26–37. 24846829 10.1016/j.jsbmb.2014.05.005

[69] Sapin E Peyron C Roche F. Chronic intermittent hypoxia induces chronic low-grade neuroinflammation in the dorsal hippocampus of mice. Sleep. 2015;10:1537–46. 10.5665/sleep.5042 PMC4576327 26085297

[70] Schmidt-Kastner R. Genomic approach to selective vulnerability of the hippocampus in brain ischemia-hypoxia. Neuroscience. 2015;309:259–79. 26383255 10.1016/j.neuroscience.2015.08.034

[71] Schwarzer C Sperk G Rauca C Pohle W. Neuropeptide Y and somatostatin immunoreactivity in the rat hippocampus after moderate hypoxia. Naunyn Schmiedebergs Arch Pharmacol. 1996;354:67–71. 8832590 10.1007/BF00168708

[72] Shao Q Liu J Li G. Proteomic analysis reveals that mitochondria dominate the hippocampal hypoxic response in mice. Int J Mol Sci. 2022;22:14094. 10.3390/ijms232214094 PMC9697535 36430571

[73] Sheldon RA Hall JJ Noble LJ Ferriero DM. Delayed cell death in neonatal mouse hippocampus from hypoxia-ischemia is neither apoptotic nor necrotic. Neurosci Lett. 2001;3:165–8. 10.1016/s0304-3940(01)01788-8 11343828

[74] Snyder B Wu HK Tillman B Floyd TF. Aged mouse hippocampus exhibits signs of chronic hypoxia and an impaired HIF-controlled response to acute hypoxic exposures. Cells. 2022;11:423. 35159233 10.3390/cells11030423 PMC8833982

[75] Solanki P Prasad D Muthuraju S Sharma AK Singh SB Ilavzhagan G. Preventive effect of piracetam and vinpocetine on hypoxia-reoxygenation induced injury in primary hippocampal culture. Food Chem Toxicol. 2011;49:917–22. 21193009 10.1016/j.fct.2010.12.015

[76] Song S Tan J Miao Y Zhang Q. Effect of different levels of intermittent hypoxia on autophagy of hippocampal neurons. Sleep Breath. 2017;21:791–8. 28553681 10.1007/s11325-017-1512-7

[77] Stroev SA Tjulkova EI Samoilov MO Pelto-Huikko MT. One- and three-time mild hypobaric hypoxia modifies expression of mitochondrial thioredoxin-2 in hippocampus of rat. Acta Neurobiol Exp (Wars). 2011;71:244–55. 21731078 10.55782/ane-2011-1844

[78] Tang W Xin X O’Connor M. Transient sublethal hypoxia in neonatal rats causes reduced dendritic spines, aberrant synaptic plasticity, and impairments in memory. J Neurosci Res. 2020;8:1588–604. 10.1002/jnr.24652 32495348

[79] Tang Z Cheng S Sun Y. Early TLR4 inhibition reduces hippocampal injury at puberty in a rat model of neonatal hypoxic-ischemic brain damage via regulation of neuroimmunity and synaptic plasticity. Exp Neurol. 2019;321:113039. 31442443 10.1016/j.expneurol.2019.113039

[80] Titus AD Shankaranarayana RB Harsha HN. Hypobaric hypoxia-induced dendritic atrophy of hippocampal neurons is associated with cognitive impairment in adult rats. Neuroscience. 2007;1:265–78. 10.1016/j.neuroscience.2006.11.037 17222983

[81] Turovsky EA Turovskaya MV Kononov AV Zinchenko VP. Short-term episodes of hypoxia induce posthypoxic hyperexcitability and selective death of GABAergic hippocampal neurons. Exp Neurol. 2013;250:1–7. 24041985 10.1016/j.expneurol.2013.09.006

[82] Vetrovoy O Sarieva K Galkina O. Neuroprotective mechanism of hypoxic post-conditioning involves HIF1-associated regulation of the pentose phosphate pathway in rat brain. Neurochem Res. 2019;6:1425–36. 10.1007/s11064-018-2681-x 30448928

[83] Vetrovoy O Sarieva K Lomert E. Pharmacological HIF1 inhibition eliminates downregulation of the pentose phosphate pathway and prevents neuronal apoptosis in rat hippocampus caused by severe hypoxia. J Mol Neurosci. 2020;5:635–46. 10.1007/s12031-019-01469-8 31865524

[84] Vetrovoy O Stratilov V Nimiritsky P Makarevich P Tyulkova E. Prenatal hypoxia induces premature aging accompanied by impaired function of the glutamatergic system in rat hippocampus. Neurochem Res. 2021;46:550–63. 33389385 10.1007/s11064-020-03191-z

[85] Vetrovoy O Tulkova E Sarieva K Kotryahova E Zenko M Rybnikova E. Neuroprotective effect of hypobaric hypoxic postconditioning is accompanied by dna protection and lipid peroxidation changes in rat hippocampus. Neurosci Lett. 2017;639:49–52. 28025115 10.1016/j.neulet.2016.12.054

[86] Vivekanandarajah A Aishah A Waters KA Machaalani R. Intermittent hypercapnic hypoxia effects on the nicotinic acetylcholine receptors in the developing piglet hippocampus and brainstem. Neurotoxicology. 2017;60:23–33. 28235547 10.1016/j.neuro.2017.02.011

[87] Wang CY Xie JW Wang T. Hypoxia-triggered m-calpain activation evokes endoplasmic reticulum stress and neuropathogenesis in a transgenic mouse model of Alzheimer’s disease. CNS Neurosci Ther. 2013;10:820–33. 10.1111/cns.12151 PMC6493504 23889979

[88] Wang H Xiong W Hang S Wang Y Zhang S Liu S. Depletion of SENP1-mediated PPARgamma SUMOylation exaggerates intermittent hypoxia-induced cognitive decline by aggravating microglia-mediated neuroinflammation. Aging (Albany NY). 2021;11:15240–54. 10.18632/aging.203084 PMC8221356 34035184

[89] Wang X Cui L Ji X. Cognitive impairment caused by hypoxia: from clinical evidences to molecular mechanisms. Metab Brain Dis. 2022;37:51–66. 34618295 10.1007/s11011-021-00796-3

[90] Wang Z Liu D Zhan J. Melatonin improves short and long-term neurobehavioral deficits and attenuates hippocampal impairments after hypoxia in neonatal mice. Pharmacol Res. 2013;76:84–97. 23917218 10.1016/j.phrs.2013.07.008

[91] Wei R Zhang R Xie Y Shen L Chen F. Hydrogen suppresses hypoxia/reoxygenation-induced cell death in hippocampal neurons through reducing oxidative stress. Cell Physiol Biochem. 2015;2:585–98. 10.1159/000430122 25997722

[92] Wu FF Zhang KL Wang ZM. Benefit of a single simulated hypobaric hypoxia in healthy mice performance and analysis of mitochondria-related gene changes. Sci Rep. 2021;1:4494. 10.1038/s41598-020-80425-8 PMC7904831 33627689

[93] Xue M Chen S Xi J. Protection against hypoxia-reoxygenation injury of hippocampal neurons by H(2)S via promoting phosphorylation of ROCK(2) at Tyr722 in rat model. Molecules. 2022;27:4567. 35889443 10.3390/molecules27144567 PMC9319530

[94] Yagishita S Suzuki S Yoshikawa K. Treatment of intermittent hypoxia increases phosphorylated tau in the hippocampus via biological processes common to aging. Mol Brain. 2017;1:2. 10.1186/s13041-016-0282-7 PMC5217192 28057021

[95] Yang W Wu W Zhao Y. Caveolin-1 suppresses hippocampal neuron apoptosis via the regulation of HIF1alpha in hypoxia in naked mole-rats. Cell Biol Int. 2022;12:2060–74. 10.1002/cbin.11890 PMC9826031 36054154

[96] Yang XH Liu HG Liu X Chen JN. Thioredoxin and impaired spatial learning and memory in the rats exposed to intermittent hypoxia. Chin Med J (Engl). 2012;17:3074–80. 22932184

[97] Yang XY Geng L Li R. Huperzine A-liposomes efficiently improve neural injury in the hippocampus of mice with chronic intermittent hypoxia. Int J Nanomedicine. 2023;18:843–59. 36824413 10.2147/IJN.S393346 PMC9942512

[98] Yu L Wan H Jin W. Protective effects of effective ingredients of Danshen (Radix Salviae Miltiorrhizae) and Honghua (Flos Carthami) compatibility after rat hippocampal neurons induced by hypoxia injury. J Tradit Chin Med. 2018;5:685–97. 32185985

[99] Zamorskii II Sopova IY Khavinson V. Effects of melatonin and epithalamin on the content of protein and lipid peroxidation products in rat cortex and hippocampus under conditions of acute hypoxia. Bull Exp Biol Med. 2012;1:51–3. 10.1007/s10517-012-1873-7 23330089

[100] Zhang H Zhang X Liu Z. Time-course effects and mechanisms of hypobaric hypoxia on nervous system in mice. Neurosci Lett. 2023;801:137163. 36868397 10.1016/j.neulet.2023.137163

[101] Zhang K Zhao T Huang X. Preinduction of HSP70 promotes hypoxic tolerance and facilitates acclimatization to acute hypobaric hypoxia in mouse brain. Cell Stress Chaperones. 2009;4:407–15. 10.1007/s12192-008-0094-5 PMC2728275 19105051

[102] Zhang K Zhao T Huang X. Notch1 mediates postnatal neurogenesis in hippocampus enhanced by intermittent hypoxia. Neurobiol Dis. 2014;64:66–78. 24368168 10.1016/j.nbd.2013.12.010

[103] Zhang Q Gao WY Zhang Y. Protective effects of astragalus extract against intermittent hypoxia-induced hippocampal neurons impairment in rats. Chin Med J (Engl). 2013;8:1551–4. 23595393

[104] Zhang Y Lan R Wang J. Acupuncture reduced apoptosis and up-regulated BDNF and GDNF expression in hippocampus following hypoxia-ischemia in neonatal rats. J Ethnopharmacol. 2015;172:124–32. 26116163 10.1016/j.jep.2015.06.032

[105] Zheng DB Lim HM Pene JJ White HR. Chicken riboflavin-binding protein cDNA sequence and homology with milk folate-binding protein. J Biol Chem. 1988;23:11126–9. 3403518

[106] Zhu D He B Zhang M. A multimodal MR imaging study of the effect of hippocampal damage on affective and cognitive functions in a rat model of chronic exposure to a plateau environment. Neurochem Res. 2022;4:979–1000. 10.1007/s11064-021-03498-5 PMC8891211 34981302

[107] Zhuravin IA Dubrovskaya NM Vasilev DS Postnikova TY Zaitsev AV. Prenatal hypoxia produces memory deficits associated with impairment of long-term synaptic plasticity in young rats. Neurobiol Learn Mem. 2019;164:107066. 31400467 10.1016/j.nlm.2019.107066

[108] Zuena AR Casolini P Venerosi A Alema GS Nicoletti F Calamandrei G. Selective reduction in the expression of type-1 metabotropic glutamate receptors in the hippocampus of adult rats born by caesarean section. Int J Dev Neurosci. 2021;81:333–41. 33759234 10.1002/jdn.10105

[109] An J. Huperzine A Improves Cognitive Dysfunction Induced by Chronic Intermittent Hypoxia in Mice and Its Related Mechanism of Iron Metabolism [Master Degree]. Hebei College of Traditional Chinese Medicine; 2020.

[110] Cao G Wang C Zhao J Duan S. Effect of CoCl2-induced hypoxia on LDHA expression in rat hippocampal neurons. J Brain Nerve Dis. 2019;4:48–752.

[111] Chen J. Expression of HIF-1α in the Hippocampus of Rats after High Altitude Hypoxia [master degree]. Qinghai University; 2022.

[112] Chen L. Experimental Study on the Effects of Chronic Hypoxia on Myelination and Function in Adult Mouse Brain [master degree]. The Chinese People’s Liberation Army (PLA) Army Military Medical University; 2021.

[113] Chen S Leng Y Zhang M Lin L Yuan L; Consolidation. Protective effect of nasal insulin on memory impairment in mice at high altitude. J Air Force Med Univ. 2023;7:1–20.

[114] Dang Y Jia S Zhao W Weijian E. Mechanism of rhodiola on hemodynamics and neuronal apoptosis in cerebral ischemia-hypoxia rats. J Brain Nerve Dis. 2021;241:27–732.

[115] Ding H. To Investigate the Mechanism of Hypercapnia Aggravating Cognitive Impairment by Promoting the Activation of NLRP3 Inflammasome in Hypoxia-activated Microglia [PhD thesis]. Southern Medical University; 2018

[116] Dong X-R Guan R-L. Mitochondrial transport protein HUMMR promotes axonal growth in rat neurons under hypoxic conditions. J Neuroanat. 2023;04:407–12.

[117] Du P. The Mechanism of lncRNA MALAT1 Regulating the Inflammatory Response of Hippocampal Neurons in Patients with T2DM and OSA [master degree]. Tianjin Medical University; 2020.

[118] Gao W Wang W-P Liu L Yang F Tu S-F. Effects of hypoxia combined with propofol on hippocampal neurons of immature SD rats through ferroptosis. J Army Med Univ. 2023;05:380–5.

[119] Han S Liu X Cui H Pan W. Effects of dihydrotestosterone on spatial learning, memory and synaptic plasticity in persistent hypoxia SAMP8 male mice. J Hebei Med Univ. 2023;07:754–9.

[120] Kong H-J Li X-L Wang F Chen X-A. Exercise preconditioning mediates oxidative stress-mediated regulation of PI3K/Akt and Nrf2/HO-1 pathways on cognitive and learning disabilities in rats. Chin J Rehabil. 2023;03:231–40.

[121] Li H Zhang Y Zhou A Wang X Dong J Liu SF. Correlation between chronic intermittent hypoxia and depressive behavior. Chin J Otolaryngol Head Neck Surg. 2021;03:17. 8–181.

[122] Li L Zhao Q He J Wang P Liu J Wang SL. Effect of TIGAR gene overexpression on hypoxia-reoxygenation injury of hippocampal neurons in rats. J Precis Med. 2018;03:230–3.

[123] Li M. To Study the Protective Effect and Molecular Mechanism of Well-point Blood-letting on Brain Injury in Acute High Altitude Hypoxia Rats based on PI3K-AKT-mTOR Signaling Pathway Mediated Mitophagy [master]. Qinghai University; 2022.

[124] Li M Wang C Tong L Shi Y Ren Y Li YP. Study on the protective effect of blood-exsanguination at well-points on acute high altitude hypoxic brain injury based on PI3K/AKT/mTOR signaling pathway. World Sci Technol. 2023;12:1–9.

[125] Li N CAI K Li W. Neuroprotective effect of sodium pyruvate on HT22 cells in mouse hippocampus. Chin Pharmacol Bull. 2023;08:1522–6.

[126] Li N Wang L Zhang K Huang M Fu J. Ginkgolide B improves cognitive impairment induced by acute hypobaric hypoxia exposure in rats and its molecular mechanism. Joint Service Military Med. 2023;01:6–10.

[127] Li X. Effects of Pioglitazone on Oxidative Stress and Cognitive Function in Intermittent Hypoxia Mice [master]. Soochow University; 2017.

[128] Li X. To Investigate the Effect of Hypoxia on PI3K/Akt/mTOR-HIF-1α Signaling Pathway in Rat Hippocampus and the Improvement Effect of Verbascoside [master degree]. Gansu University of Traditional Chinese Medicine; 2020.

[129] Li X. Effects of Tetramethylpyrazine Hydrochloride on Cognitive Function and GABAR Expression in Hypobaric Hypoxia Rats [master]. Qinghai University; 2018.

[130] Li X Zhang Z Li X. Role of adenosine A_(2A) receptor in spatial memory impairment in chronic intermittent hypoxia mice. J Med Res. 2023;05:28–33.

[131] Mechanism of Oxidative Stress in Cognitive Impairment and Hippocampal Damage Induced by Intermittent Hypoxia in Young Rats [Dr]. Lanzhou University; 2020.

[132] Liu F. To Investigate the Role and Mechanism of NF-κB-Mediated JNK Signaling Pathway in Cognitive Dysfunction in a Rat Sleep Apnea Model [PhD]. China Medical University; 2018.

[133] Liu J. Metabolomics of Hippocampus and Serum in Hypoxic Preconditioned Mice [Master]. The Third Military Medical University; 2021.

[134] Liu P Hu Y Jiang J Zhao S Deng H Xue X. Effects of Tongxinluo capsule on inflammatory response, brain tissue edema and cognitive function after hypobaric hypoxia exposure in rats. Chin J Comparative Med. 2021;06:69–76.

[135] Liu X-Q. To Study the Mechanism of Hypercapnia Aggravating the Destruction of Blood-brain Barrier by Promoting the Nuclear Translocation of Hypoxia-inducible Factor-1α Subunit of Astrocytes in Hippocampus in Rats with Hypoxemia [Dr]. Southern Medical University; 2019.

[136] Liu X. Effects of Dihydrotestosterone on Spatial Learning, Memory and Synaptic Plasticity in Hippocampus of SAMP8 Male Mice under Continuous Hypoxia [Master degree]. Hebei Medical University; 2021.

[137] Qi R Li N Wang L. Effects of hypoxic preconditioning on mitochondrial energy metabolism in mouse hippocampal HT22 cells. J Anhui Med Univ. 2022;57:Note: 85-1588.

[138] Qiao H. To Study the Myelin Damage and Repair Mechanism of White Matter Fiber Tracts in Mice under Hypoxia/Reoxygenation [Master]. Xiamen University; 2019.

[139] Qin N Shen Y Cheng J Zhang X Li W Wang R. Protective effect of salidroside on memory impairment in mice under high altitude hypoxia. Chin Pharmacol Bull. 2023;01:6 5–70.

[140] Shi H. Study on the Mechanism of Brain Injury induced by Acute High Altitude Hypoxia Combined with Sodium Cyanide Poisoning in Mice [Master Degree]. Tianjin University; 2021.

[141] Shi Z Hu Y Ruan Q Fan M Zhao M Zhu L. Hypoxia promotes CXCL10 expression in microglia induced by lipopolysaccharide. Acta Physiol Sin. 2023;02:153–9. 37089089

[142] Sun Y Zhai M Tan Z. Protective effect of ISRIB on neuronal dendritic spine morphology under high altitude hypoxia exposure. J Air Force Med Univ. 2022;07:685–9.

[143] Thomas. Effects of Excitation or Inhibition of Basal Forebrain Cholinergic Neurons on Cognitive Performance in Mice Exposed to Chronic Intermittent Hypoxia [Dr]. Wuhan University; 2020. 10.1016/j.brainresbull.2020.08.027 32905806

[144] Wan Y. To Explore the Neuroprotective Effect of Hypobaric Hypoxia Preconditioning on Cerebral Infarction in Rats and Its Mechanism [Dr]. Qinghai University; 2022.

[145] Wang H. The Regulatory Mechanism of SENP1 on Chronic Intermittent Hypoxia-Induced Neuroinflammation and Cognitive Impairment in Mice [Dr]. Southern Medical University; 2021.

[146] Wang L Yang F Huang C. To investigate the effect of edaravone on autophagy and apoptosis in brain tissue of rats after intermittent hypoxia injury. Chin J Gerontol. 2023;03:660–4.

[147] Wang L Zhang P Fu A Wang Y Huang C. Effects of intermittent hypoxia on the expression of synapse-associated proteins in hippocampal neurons and cognitive function in rats. Chin J Cardio-Cerebrovasc Dis. 2020;05:534–7.

[148] Guo MD. Mechanism of Euphorbian-Jiannao decoction mediating CREB/BDNF pathway to regulate synaptic plasticity of hippocampal neurons in rats under low glucose and hypoxia conditions. Chin J Basic Med Tradit Chin Med. 2022;09:1433–8.

[149] Wang X Ren J Li J Liu H Hu Z. Mechanism of P2X7 receptor on cognitive dysfunction in rats with obstructive sleep apnea hypopnea syndrome. South China J Def Med. 2022;03:163–7.

[150] Weng QM Wang M Xie W Xie YB. The expression of miR-103 and its target gene Cav1.2 in hypoxic preconditioning to promote learning and memory in mice. J Baotou Med Coll. 2023;06:10–4.

[151] Xiong Yang. To Analyze the Correlation between Hippocampal Metabolism and Cognitive Impairment in OSA Patients [Master]. Shanxi Medical University; 2022.

[152] Xu X Xu L Wang Y. Effects of remote ischemic postconditioning on tissue plasminogen activator/brain-derived neurotrophic factor pathway and hippocampal synaptic plasticity in neonatal hypoxic-ischemic encephalopathy mice. Chin J Ophthalmol. 2022;04:296–300.

[153] Xu Y Li X Shan L. Changes in glutamate levels in the cerebellum and hippocampus of obstructive sleep apnea-hypopnea syndrome animal model rats. Chin J Med Front. 2018;10:The word - 28.

[154] Xu Yong. Dexmedetomidine Alleviates Necroptosis Mediated Hypoxia-Induced Injury and Inflammation in HT22 Cells [master degree]. South China University; 2019.

[155] Yan J Hou J Ren X Luo H Sheng Y Wang BT. Molecular mechanism of hippocampal mitochondrial damage and base excision repair in intermittent hypoxia rats. 5th West China Sleep Medicine Congress and Chongqing Mental Health Association Sleep Medicine Academic Annual Meeting, Chongqing, China; 2018

[156] Yang J Hou B Wu W Yan X Hao A Pang YQ. Protective effects of hypoxic preconditioning on hippocampal neurons in mice and related genes. Chin J Med. 2023;04:32–7.

[157] Yao B Song W Xu Z Zhu J. Effects of propofol on cognitive function in chronic intermittent hypoxia mice and its mechanism. J Shanxi Med Univ. 2022;07:849–54.

[158] Zhang C. mTOR/NF-κB Pathway is Involved in the Regulation and Mechanism of BDNF-Mediated Synaptic Plasticity in Primary Hippocampal Neurons of Rats Under Chronic Intermittent Hypoxia. Shandong University; 2021.

[159] Zhang F. To Investigate the Pathological Effects and Mechanisms of Chronic Sleep Deprivation and Acute Hypoxia on Alzheimer’s Disease. Dalian Medical University; 2018.

[160] Zhang P Han X Wang Y Huang C Wang H. Effects of cistanche glycosides on oxidative stress and cognitive function in intermittent hypoxia rats. Chin J Respir Crit Care. 2023;04:26 9–273.

[161] Zhang S. The role and mechanism of ATP-sensitive potassium channels in mitochondrial inner membrane in the induction of cerebral ischemic tolerance by chronic intermittent hypobaric hypoxia preconditioning. Hebei Medical University; 2017.

[162] Zhang W Song Y Yang J. Effect of butylphthalide preconditioning on memory function and acetylcholine content in hippocampus of acute hypobaric hypoxia mice. J Integr Tradit Chin West Med Cardiocerebral Vasc Dis. 2018;8:But be 59-1661.

[163] Zhao Z Zhou Y Dong X Cao Z. Curcumin improves learning and memory impairment and synaptic damage in the hippocampus of hypoxia-induced mice by metabolomics. The Chinese Society of Toxicology the 10th National Conference on Toxicology. Vol. 5. 2023:11.

[164] Zhong Ting. Protective Effect of Ketogenic Diet on cognitive dysfunction in Mice Exposed to High Altitude Hypobaric Hypoxia [master degree]. Southwest University of Medical Sciences; 2020.

[165] Zhou Y Cao Z Chen X Li M Zhan J. Effects of hypoxia on the expression of molecules related to the development of hippocampal neurons in mice. Chin J Neuroanat. 2022;04:417–22.

[166] Zhou Y Zhao Z Shen X Luo W Cao Z. Effects of chronic hypobaric hypoxia exposure on dendritic spine morphology and filamin A expression in hippocampal CA1 neurons of mice. Chin J Physiol. 2018;11:Just as 09-2214.

